# Collagen-Based Wound Dressings: Innovations, Mechanisms, and Clinical Applications

**DOI:** 10.3390/gels11040271

**Published:** 2025-04-05

**Authors:** Adina Alberts, Andreea Gabriela Bratu, Adelina-Gabriela Niculescu, Alexandru Mihai Grumezescu

**Affiliations:** 1 Carol Davila University of Medicine and Pharmacy, 050474 Bucharest, Romania; adina-magdalena.alberts@rez.umfcd.ro; 2 Faculty of Chemical Engineering and Biotechnologies, National University of Science and Technology Politehnica Bucharest, Gh. Polizu St. 1-7, 060042 Bucharest, Romania; andreea.bratu2910@stud.fim.upb.ro (A.G.B.); adelina.niculescu@upb.ro (A.-G.N.); 3 Research Institute of the University of Bucharest—ICUB, University of Bucharest, 050657 Bucharest, Romania

**Keywords:** collagen, collagen wound dressing, chronic wounds, wound healing, angiogenesis, polymers, crosslinking

## Abstract

Collagen-based wound dressings have developed as an essential component of contemporary wound care, utilizing collagen’s inherent properties to promote healing. This review thoroughly analyzes collagen dressing advances, examining different formulations such as hydrogels, films, and foams that enhance wound care. The important processes by which collagen promotes healing (e.g., promoting angiogenesis, encouraging cell proliferation, and offering structural support) are discussed to clarify its function in tissue regeneration. The effectiveness and adaptability of collagen dressings are demonstrated via clinical applications investigated in acute and chronic wounds. Additionally, commercially accessible collagen-based skin healing treatments are discussed, demonstrating their practical use in healthcare settings. Despite the progress, the study discusses the obstacles and restrictions encountered in producing and adopting collagen-based dressings, such as the difficulties of manufacturing and financial concerns. Finally, the current landscape’s insights indicate future research possibilities for collagen dressing optimization, bioactive agent integration, and overcoming existing constraints. This analysis highlights the potential of collagen-based innovations to improve wound treatment methods and patient care.

## 1. Introduction

The skin represents the human body’s largest organ and is an essential barrier in maintaining internal equilibrium and defending against exterior risk factors [1]. Regular exposure to external factors like burns, cuts, toxic substances, and infections makes the skin vulnerable to several wounds [2,3]. Wound healing represents a complex and dynamic physiological process; improper or delayed treatment can result in chronic wounds. Several crucial characteristics, including the anatomical placement of the wounds and complications induced by concurrent disorders that the patients may have, influence the complexity of chronic wounds [4,5,6]. The wound healing physiological process consists of four steps that occur chronologically but overlap: hemostasis, inflammation, proliferation, and remodeling [7,8]. The Wound Healing Society categorizes chronic wounds into four types based on their causes: pressure ulcers (PUs), diabetic foot ulcers (DFUs), venous leg ulcers (VLUs), and arterial insufficiency ulcers. Chronic wounds significantly affect the healthcare system due to their rising prevalence and associated costs. This can cause concerns when all viable treatment options have been attempted and amputation is required. Ulcers are responsible for 85% of all amputations, with DFU accounting for 70% of all lower limb losses. Amputations are performed every 30 s globally due to non-healing DFU [5,9,10,11].

Most chronic wounds are linked to illnesses that are more frequent in the elderly than in young people, such as diabetes mellitus, vascular insufficiency, malnutrition, cancer, skin conditions, obesity, autoimmune disorders, and hypertension. Fundamental issues remain concerning how aging affects wound repair and the mechanics of wound healing and tissue regeneration in older people [12,13,14]. In the United States, 3% of people over the age of 65 suffer from open wounds. The U.S. government predicts that the number of seniors will be around 95 million by 2060, indicating that chronic wounds will represent a significant issue in this demographic. Chronic wounds cause harm globally and have a negative global impact. The global advanced wound care market is expected to reach USD 18.7 billion by 2027, with a compound annual growth rate (CAGR) of 6.6% from 2020 to 2027 [15,16].

Traditional dressings, including gauze, cotton wool, lint, and natural or synthetic bandages, are commonly used for skin wounds. Their primary role is to protect the wound, enabling wound exudates to evaporate and avoiding bacterial infection. However, they primarily provide mechanical protection and have limitations such as wound exudates’ restricted absorptive ability and frequent replacements’ burden [17,18,19,20,21].

The development of innovative multifunctional wound dressings that suit all the needs of skin wound healing is urgently required. Several important criteria for optimal wound dressings have been suggested: (1) low immunological response, non-toxicity, and superior histocompatibility; (2) lasting preservation of moisture for maintaining a humid environment for the wound surface; (3) adequate mechanical and physical durability to ensure the integrity of the dressing; (4) outstanding antibacterial properties to prevent subsequent infection of the wound; (5) higher permeability that enables gas and water vapor interaction with the external environment; (6) non-adhesion that allows for the harmless removal of worn dressings [19,22,23,24,25].

Collagen represents a fundamental component of the extracellular matrix (ECM) essential to sustain the skin’s structural integrity and control wound healing stages in its fibrillar or soluble state. Collagen has the primary benefit of having minimal antigenicity, excellent biodegradability, biocompatibility, and being easily accessible and highly adaptable [26,27]. Collagen-based dressings have demonstrated great promise for tissue engineering and regenerative medicine. Furthermore, numerous studies have been conducted on developing collagen-based drug delivery systems, which immobilize proteins and small molecules to regulate their release for use in biomedical applications. These methods protect the loaded molecules’ biological function while limiting the release from collagen scaffolds, resulting in ideal effects on cells of interest [28,29].

A comprehensive analysis focusing on numerous characteristics of collagen dressings might bring novel insights into regenerative medicine and tissue engineering. The following review aims to provide an overview of current advancements in collagen dressings for wound healing. Initially, biology, key properties, and mechanisms of collagen in wound healing were introduced. Second, many types of collagen dressings and their manufacturing techniques were emphasized. After that, the innovations in the functionalization of collagen-based dressings have been explored, along with clinical applications across different types of wounds, challenges, and limitations. Lastly, commercial collagen dressings have been revised, and future perspectives target integrated smart materials, bioactive compounds, and individualized wound care approaches.

## 2. Composition, Types, and Sources of Collagen

Mammals’ ECM is mostly composed of collagen. It is the primary protein of connective tissue and constitutes around 25% of all human proteins. Originally described as “that part of connective tissue that produces gelatin on boiling”, the French word “collagen” and the Greek words “kolla” (glue) and “gen” (producer) were used to refer to the glue-producing component of connective tissue [30,31,32].

Collagen exists in many different forms throughout nature. A right-handed triple helical structure is formed by three polypeptide molecules, known as “α chains”, each of which is a left-handed chain. The molecule can either be a heterotrimer, in which one or more of the α chains are typically different from one another, or a homotrimer, in which all the α chains are identical. Tropocollagen is the name given to each helical structure. The repeating amino acid pattern is Gly-X-Y. Gly represents glycine, and X or Y can be any amino acid, usually hydroxyproline or proline, which is present in every polypeptide chain and is commonly known as procollagen. The X and Y amino acids are visible at the protein’s surface, whereas glycine is at its center [33,34].

**Figure 1:**
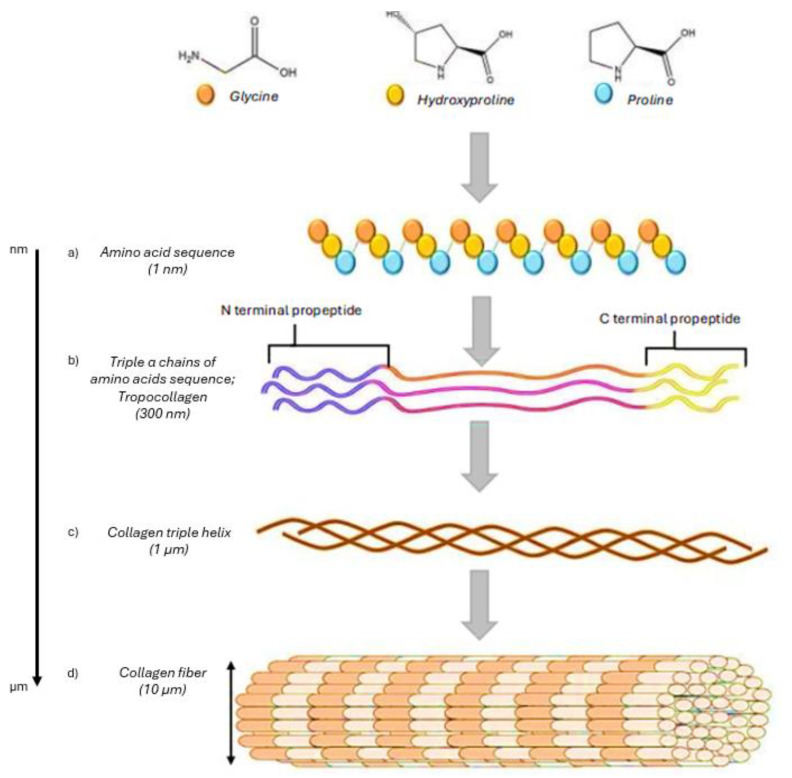
Illustration of collagen’s structure: (**a**) amino acid sequence, (**b**) tropocollagen, (**c**) collagen triple helix, and (**d**) collagen fiber. Adapted from open-access sources [38,39].

Figure 1 illustrates current knowledge about the intracellular processing of collagen molecules, the assembly of the triple helix, and the export of procollagen, which continues to be converted to collagen extracellularly. All fibrillar collagens are initially produced in their native intracellular environment as precursor molecules, which include large amino- and carboxy-terminal propeptides. These precursor molecules are then directed to the rough endoplasmic reticulum (RER), where post-translational modifications take place, and procollagen molecules are assembled. Lysyl and prolyl hydroxylase enzymes in the RER lumen hydroxylate specific lysine and proline residues in the collagen propeptides to enhance the triple helices’s stability after formation, producing a mechanically stable fibril [32,34,35]. The association of the C-propeptide domains initiates triple helix trimerization. This leads to the joining of α-chains to form procollagen after lysyl and prolyl hydroxylation and O-linked glycosylation. The Golgi apparatus packages procollagen for release into the extracellular matrix (ECM). Mature collagen molecules are generated by proteolytic processing of the major NH_2_^−^ and COOH^−^ propeptide domains following or during procollagen secretion. These molecules then self-assemble into fibrils with widths ranging from 0.5 to 3 μm. Furin-like proprotein convertases and collagen-type-specific matrix metalloproteinases (MMPs) contribute to the propeptide domains’ enzymatic removal. The N-propeptide’s persistence affects the fibril’s diameter and shape without influencing fibril development. Meanwhile, retaining the C-propeptide leads to high procollagen solubility in the extracellular space, which prevents premature fibril assembly. After collagen fibril aggregates are formed, further modifications occur to create mature collagen fibers. Covalent crosslinks are introduced to the supramolecular assembly in the final stage of collagen production to enhance stability and improve mechanical properties [32,34,36,37].

Currently, 28 types of collagens have been identified. The supramolecular structure and organization allow them to be classified as follows: anchoring and transmembrane fibril collagens, basement-membrane collagens, collagens formed by NETs (network-forming), fibril-forming collagens, fibril-bound collagens (Fibril Associated Collagens with Interrupted Triple Helices, or FACIT), and others with distinct characteristics (Table 1) [38,40].

Various collagen types’ structure, assembly, and function are distinguished by their considerable complexity and variety, including nonhelical domains, extra connections, and variations in their linkage. Collagen-forming fibrils are the most common and abundant family of collagens, accounting for over 90% of all collagens. Type II and XI collagens primarily belong to the filamentous matrix articular cartilage, whilst types I and V collagen fibrils help to form the bone spine. Their tissues are stable and intact because of their extensibility strength and torsional stability, preventing bone fractures. Type IV collagens have a more flexible triple helix joined into a mesh and are restricted to foundation membranes (for digestion or respiration). The strongly crosslinked disulfide is a type VI collagen-forming microfiber. Collagens linked to FACIT are also recognized. The IX, XII, and XIV collagens are part of this type of collagen and help control the diameter of collagen fibers. Collagens VIII and X are made up of hexagonal lattices, whilst collagens XIII and XVII incorporate cell membranes [38,43].

Recently, the significant regulatory roles of the matrisome (the assembly of ECM proteins and related factors) have highlighted the different physiological roles of collagen that go together with its mechanical properties. Considering the specificity of binding displayed by several receptors, structural variations in collagen originating from different animal and plant species will result in functional modifications in the interaction of collagens. Since collagen type I is affordable and relatively abundant, it is the main form utilized in the scientific field. The most prevalent collagen type I is generated from rat tail tendon, porcine, and bovine skin. Options like human placenta and human-skin-derived collagen type I are more expensive. While recombinant and marine-derived sources have been employed, collagen type III has historically been obtained from human, bovine, and chicken skins as well as human placentas [44,45].

Bovine collagen is generated from cow skin and bone and is the main source of collagen in the industry. Researchers are looking for a safer alternative supply of collagen because of the rise of illnesses that are harmful to humans, including bovine spongiform encephalopathy (BSE), transmissible spongiform encephalopathies (TSE), and foot-and-mouth disease (FMD), suggesting that source population and purifying techniques are crucial factors. Bovine collagen has good biocompatibility and low immunogenicity—it is generally readily accepted in vivo and does not trigger an immunological response in most people, except for those who have a severe collagen allergy [46]. Consistency varies according to genetic inheritance, age, and place of origin. The age of bovine tissue affects how much collagen is recovered after isolation as younger tissues produce more collagen. Collagen type I is manufactured in an industrial setting from the Achilles tendon of cows. Type II comes from nasal or articular cartilage, whereas type IV comes from the placental villi. The bovine dermis is utilized in different phases of development, including fetal and neonatal stages for tendon reinforcement, wound healing, and skin regeneration (via collagen matrix), hernia restoration, and reconstructive and plastic surgery, as well as adult pericardium for strengthening muscle flaps [47,48,49].

In contrast to industrial use, the rat-tail tendon (RTT) constitutes one of the most utilized sources of collagen type I among scientists due to the large body of literature about isolation and characterization. By weight, RTT comprises 90–95% collagen type I; this high amount guarantees notable yields following isolation. According to research, the rat’s collagen gets less flexible and more force-resistant as it ages. Collagen scaffolds made from crosslinked RTT have a significantly greater tangent modulus than scaffolds made from non-crosslinked collagen. The age of the source rat population is predicted to affect the crosslink density and scaffold mechanical characteristics as keto-imine crosslinks resist acid extraction more than aldimine linkages. Despite being extensively utilized in research, RTT collagen type I is not used in clinical products due to the lack of medical-grade RTT collagen I [48,50].

Collagen is found in a variety of marine creatures, including sponges (Porifera), jellyfish (Cnidaria), fish (Pisces), animals (mussel, squid, cuttlefish, and octopus), and certain echinoderms. Fish collagens are beneficial due to the lower risk of disease transmission, reduced quantity of wasted collagen-containing materials in the food sector, and no religious prohibitions. The fishing industry may use leftovers from fish processing to produce affordable collagen. Collagen is abundant in bones, skin, and scales, which account for around 30% of this waste and may thus be utilized to produce collagen. Fish collagen has greater bioavailability compared to collagen from pigs and cows and is absorbed up to 1.5 times more effectively into the body. The chemical and mechanical characteristics of collagen derived from marine sources differ from those derived from mammals. These differences include a lower melting point and viscosity at a specific concentration in solution, less water solubility, more substantial fibrillar proportions of alanine and glutamic acid, and a lower proportion of proline. In terms of tissue engineering and biomaterials, the biological characteristics of collagen derived from marine sources are also very advantageous; scaffolds made of collagens isolated from marine sources exhibit high biocompatibility, low immunogenicity, and high biodegradability, which permits the body to gradually replace the scaffold with regenerated tissue [48,51,52,53].

Recombinant human collagen (RHC) lacks animal components, minimizing the danger of interspecies variance and harmful infections. This ensures homogeneity and suggests a viable production technique. Because of its limited quantity or ethical constraints, it can supply amounts of collagen that are challenging to separate from tissues. Recombinant human collagen is frequently utilized in cosmetics and tissue engineering due to its cost-effective manufacturing, uniform quality, and traceability. It has been produced using yeast, transgenic tobacco, E. coli, insect cells, silkworms, mice, and transfected mammalian cells. Recombinant collagen can be produced by employing multi-gene expression techniques to precisely replicate the collagen DNA sequences in yeast or plant (or insect) cells. Collagen genes and cDNAs for prolyl hydroxylase subunits are co-expressed to produce fully hydroxylated, thermostable collagens. Chemical extraction can irreversibly break collagen crosslinks, reducing its biological function. The complete triple-helical shape of the collagen obtained by recombinant technology is crucial to the biological activity of collagen [54,55,56].

Regulatory authorities now primarily target native collagens, which differ from recombinant Col, in their procedures for assessing the safety and effectiveness of collagen-based devices. For instance, most recombinant Col are smaller molecules than native collagens and may be dissolved in water before being further filtered to sterilize. In contrast, natural collagens are biomacromolecules often insoluble in neutral solutions and cannot undergo filtering to sterilize. Collagenase is used in sodium dodecyl sulfate-polyacrylamide gel electrophoresis (SDS-PAGE) to test the purity of animal collagen. This method is specific for native collagen but may not be appropriate for recombinant Col. Consequently, recombinant Col dressings require scientific assessment techniques. The material, structure, properties, and performance characterization techniques of recombinant Col and recombinant Col dressings must be assessed and standardized scientifically. The successful transfer of recombinant Col dressings from lab to clinic depends heavily on standards and guidelines, a regulatory science component. The study of recombinant Col dressings is anticipated to yield new regulatory instruments and techniques for assessing their efficacy and safety [57].

## 3. Properties of Collagen—Suitability for Wound Dressings

Collagen is a natural protein present throughout the body, making it extremely biocompatible. Because collagen dressings have chemotactic effects on wound fibroblasts, they promote the deposition and arrangement of newly produced collagen, fostering a healing environment. Collagen-based biomaterials attract and activate certain cells throughout the healing cascade, including fibroblasts and macrophages, to improve and impact wound healing. Depending on how they are delivered, these biomaterials can either absorb or give moisture. Collagen dressings are conformable and straightforward to apply and remove, which may aid the process of wound healing. Collagen displays high biocompatibility and biodegradation to be used as a transferable scaffold for tissue reconstruction. Cell culture tests have already shown encouraging outcomes regarding cell adhesion, viability, and proliferation [37,58]. For starters, collagen can significantly lower elastase levels in a wound setting, which breaks the chronicity looping process. Furthermore, collagen’s ability to bind MMP is beneficially disruptive to chronic wounds that have biochemically stopped. All collagen-based products’ in vitro tests readily reveal this MMP-binding ability. Several polymers made from collagen have been employed as wound dressings in recent years. Denatured collagen, or collagen that has mostly lost its triple helix basic unit structure, typically makes up most products produced from collagen sources that underwent prolonged and severe processing. Collagen and other hydrophilic polymers with a high water content comprise hydrogels, which are three-dimensional crosslinked networks of polymers. Because they have structural similarities with the ECM, they are biocompatible. Hydrogels preserve the moist wound environment by supplying water molecules to the wound [58,59,60].

Collagen-based scaffolds’ mechanical properties might vary depending on factors such as collagen supply, gelation process, crosslinking agents, testing circumstances, and additives in the composite. Acid-solubilized collagen gels at 37 °C increase the assembly development rate and reduce collagen bundling (small fibrils and fibril width). This results in less order, which lowers the mechanical integrity. The amount of collagen employed also affects the fibrils’ length and diameter. The collagen concentration in the connective tissues in vivo is between 30 and 40 mg/mL, but most collagen scaffolds exhibit a concentration of 6 mg/mL or less. Collagen-only scaffolds are unsuitable for in vivo implantation due to the solubilization process’s loss of in vivo-like structural organization and diminished mechanical integrity, requiring chemical and physical changes. Collagenases, gelatinases, and nonspecific proteinases found in vivo degrade such collagen-only scaffolds quickly by hydrolysis and enzymatic destruction [36,61,62].

The scaffold structure plays a significant part in reflecting the desired response from cells through the transmission of relevant biochemical signals. Native bone is a strong composite made up of in-line, compliant collagen-type I fibrils and stiff inorganic platelets impregnated with hydroxyapatite. In the conventional collagen construction process, incorporating carbon nanotubes and inorganic materials such as calcium phosphate, hydroxyapatite (HAp), and bioglass results in scaffolds with improved modulus and strength and adjustable mineral: organic component ratios. The inclusion of these materials results in composite scaffolds with collagen as the matrix and different elements as fillers. According to one study, adding HAp may raise collagen’s modulus from 92 kPa to 209 kPa. Depending on the weight percentage of HAp, testing settings, crosslinking, and testing techniques, adding HAp can increase the collagen scaffold’s modulus by 0.1 to 89,500 kPa. Adding nanosized bioglass to plastically crushed dense collagen scaffolds improves compressive modulus compared to neat collagen scaffolds [63,64]. The collagen scaffolds are filled with polymers like glycosaminoglycans (GAGs), hyaluronan, elastin-like polypeptide (ELP), and chitosan (CS). It has been demonstrated that adding ELP to the collagen matrix strengthens the scaffold, as highlighted by a threefold rise in the modulus. Composites containing these components have lower water content, making them stiffer and more deterioration-resistant. In comparison to ELP-collagen scaffolds (22.0 kPa), the hydrated ELP–carbon nanotube–collagen (39.0 kPa) and ELP–bioglass–collagen (41.1 kPa) composites have an elastic modulus that is 1.5–2.0 times higher. According to Gurumurthy et al. [65,66], physical involvement and chemical intermediate bonding interactions among the collagen fibers and the other additional polymers cause these increases in the mechanical characteristics (strength and modulus).

Crosslinking, which generates links between individual collagen molecules, is frequently utilized to improve the mechanical characteristics of collagen-based biomaterials. By keeping the long, rod-like molecules from slipping beyond one another under stress, collagen’s abundant intramolecular and intermolecular crosslinks provide its structural strength and durability in the biological environment. Chemical crosslinking can be accomplished by employing several kinds of isocyanates, aldehydes, and carbodiimides as crosslinking agents. Dehydrothermal treatment (DHT) is the most popular physical crosslinking approach, which does not require a chemical crosslinking agent. The DHT technique uses condensation between the carboxyl and amine groups of nearby amino acid side chains to produce crosslinks. The scaffold is exposed to temperatures between 100 and 120 °C while under high vacuum, which causes the scaffold to get dehydrated to less than 2 wt.%. By altering the length of treatment, the collagen scaffold’s stiffness may be customized [67,68]. Tensile or compressive tests can be performed on the scaffolds in wet and dry conditions. Divakar et al. [69] carefully assessed the microstructure, mechanical characteristics, and processing relationships of freeze-cast dry and moist collagen films in longitudinal and radial orientations. Even though the films demonstrated a higher yield strength and modulus while assessed in the direction parallel to that of the ice growth, their mechanical characteristics indicated a more complex relationship with the cooling rate used for freeze-casting because of the various microstructures (number, size, and pore distributions) that formed.

Collagen breakdown is linked to angiogenesis, inflammation, and re-epithelialization, all of which are regulated by intricate molecular mechanisms. Soluble collagen fragments draw immune cells, such as macrophages, during the inflammatory phase, and these cells patrol the site to remove devitalized tissue and pathogens [70,71]. This helps the transition to the proliferative stage. Collagen fragments serve as potent angiogenic signals during this phase, encouraging the development of new blood vessels. Additionally, collagen encourages keratinocyte migration, which facilitates the re-epithelialization of wounds. Both intracellular and extracellular processes regulate degradation. The extracellular process involves proteolytic enzymes secreted and binding to membranes [72,73,74]. Through phagocytosis, macropinocytosis, or endocytosis, whole collagen fibrils and fragmented collagen are internalized and then broken down by enzymes in the intracellular route.

Repaired tissue remodeling is guided by proteolytic enzymes’ function at different stages of tissue healing [75]. MMPs that are crucial for collagen turnover throughout the healing of different tissues include collagenases and gelatinases, which break down intact and degraded fibrillar collagen, respectively. While MMP-9 (gelatinase) breaks down collagen IV, MMP-1 (also called collagenase-1) and MMP-8 (collagenase-2) selectively cleave collagens I and III. Numerous studies have shown that collagenolytic enzymes can identify, attach, unwind, and destroy the triple helix’s component strands. Collagen’s primary and super-secondary structures are believed to be responsible for this high specificity. Both pathological (tumorigenesis and metastasis) and physiological (the growth and tissue repair) processes are attributed to MMPs. Additionally, they facilitate the release of bioactive fragments from full-length collagens, such as tumstatin and endostatin, also called matricryptins. These components restore tissue architecture during healing by accurately directing blood channel pruning [76,77].

## 4. Biological Functions of Collagen in Wound Healing

**Figure 2:**
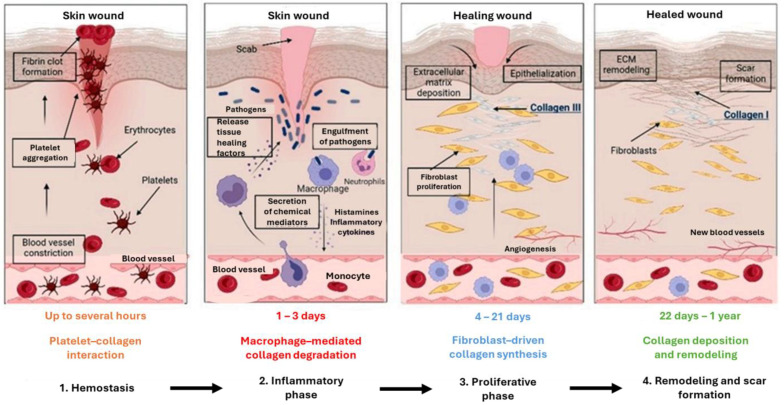
Collagen’s involvement in the wound-healing mechanism. Adapted with permission from [56]. Copyright © 2022, Elsevier.

Wound repair is a complex process divided into four steps: hemostasis, inflammation, proliferation (cellular infiltration, angiogenesis, and re-epithelialization), and maturation/remodeling. Figure 2 depicts the wound-healing process, which includes a vascular response to limit the loss of blood, the release of inflammatory agents to control bleeding and infections, the formation of new layers, and the progression of healing. These steps occur in a chronological sequence but overlap. Important phases of wound healing, including angiogenesis, inflammation, and hemostasis, are influenced by collagen, the ECM, and its constituents [78,79,80,81].

The first step is hemostasis, which occurs when bleeding stops and is followed by overlapping processes, such as inflammation, the proliferation of cells, blood coagulation, and scar formation. Hemostasis typically takes a few hours to complete and usually occurs hours after an accident. Tight constriction of injured blood vessels and blood clot formation prevent exsanguination due to vascular injury. When certain integrin receptors recognize extravascular collagen (type I collagen), platelets become activated. Research indicates that platelet receptors, including glycoprotein VI, engage with ECM proteins like collagen and fibronectin. This interaction triggers the release of growth factors, adhesive glycoproteins, and soluble mediators such as cyclic AMP, facilitating platelet adherence to the blood vessel wall [82]. Growth factors are released, and neutrophils and monocytes are recruited to initiate the inflammatory response. At the same time, endothelial cells are stimulated to begin angiogenesis. After being recruited and activated by platelet-derived growth factor (PDGF), fibroblasts migrate to the wound site and generate collagen and GAGs, facilitating cellular migration. The second inflammatory response stage begins when the bleeding stops and typically lasts 24 to 72 h following the injury. However, it can persist for five to seven days after an incident. The uneven distribution of MMPs and tissue inhibitors of metalloproteinases (TIMPs) further prolongs the inflammatory phase, delaying the formation of new collagen fragments. Neutrophils, monocytes, and macrophages clear debris and infection from the wound while releasing soluble mediators, including proinflammatory cytokines and growth factors. These help recruit and activate fibroblasts and epithelial cells as they prepare for the next healing phase. Cytokines help reduce inflammation and promote wound healing. [56,83,84].

During the proliferative healing phase, fibroblasts, keratinocytes, macrophages, and endothelial cells are triggered to mange angiogenesis, matrix deposition, and wound closure. The tissue’s function and structure are restored during this phase when new collagen fibers, proteoglycans, and fibronectin matrix replace the temporary fibrin matrix. Large quantities of type III collagen and other ECM proteins are produced. The initial processes involve blood clotting, the formation of a transient ECM, and the migration of fibroblasts and keratinocytes into the injured area to rebuild the protective barrier. There is an increase in various growth factors and other proteins, including collagen type I. In the remodeling stage, fibroblasts are activated to form mature collagen fibrils by substituting fibronectin, hyaluronan, and proteoglycans for the initial fibrin clot. Collagen type I gradually replaces collagen type III as the scar heals, enhancing the scar’s tensile strength [26,74,83]. Elastin (EL), another vital dermal ECM component, rebuilds the skin’s elastic fibers to maintain flexibility. Crosslinking is employed to synthesize it from the precursor tropoelastin. The release of EL segments, also known as elastokines, occurs when the normal dermal matrix breaks down, activating tropoelastin production rapidly. EL facilitates protease synthesis, matrix formation, tissue recoil, and cell migration. Its cell-interacting domains enhance cell adhesion, expansion, proliferation, and signaling, contributing to wound healing and skin homeostasis. Achieving complete physiological recovery may take months to years, during which further treatment is unnecessary [85,86].

Collagen promotes cell migration, differentiation, and proliferation, facilitating wound healing. Tissue remodeling and scarring are physiological processes that maintain tissue continuity. Beyond regulating fibroblast growth and proliferation, collagen contributes to wound healing and affects keratinocyte migration, attachment, differentiation, and proliferation. The beneficial properties of collagen make it a common component in tissue engineering and wound healing applications. The deposition of fibrin clots results from platelet activation and aggregation in response to an injury. Immune cell activation triggers the production of pro-inflammatory cytokines throughout the inflammatory phase and stimulates the migration of fibroblasts, epithelial cells, and endothelial cells [87,88]. The duration of tissue recovery depends on the balance between collagen synthesis and breakdown. Collagen production, deposition, and degradation are essential processes in wound healing. Basic fibroblast growth factor, tumor necrosis factor, transforming growth factor-β, platelet-derived growth factor, and interleukin-1 all promote collagen production at the injury site—the presence of fibroblasts aids in collagen deposition. Collagen breakdown releases fragments, which enhance fibroblast proliferation and stimulate the production of growth factors that support angiogenesis and re-epithelialization. Finally, the acquisition of tensile strength is determined by remodeling the ECM (the balance between matrix metalloproteinase activity and new matrix synthesis). [26,56,89,90,91].

## 5. Types and Variations of Collagen-Based Wound Dressings

Biomaterials composed of Col come in various forms and have applications in regenerative medicine, tissue engineering, and pharmacy. Collagen (Col) wound dressings can be categorized into several types based on their scaffold material compositions, including films, sponges, hydrogels, nanofibers, and powders. Each type serves distinct purposes and is tailored for specific wound management needs. Single- or multi-approach processing methods produced complex structures like double- or triple-layered scaffolds or bioactive coatings. Thus, selecting the appropriate collagen wound dressing is crucial as it directly impacts the healing process [92,93,94].

### 5.1. Films

Col films have traditionally been a barrier in wound repair and tissue engineering. Col solutions can be cast into approximately 0.1–0.5 mm thick films and air-dried as ophthalmological screens. The gradual release of encapsulated medications is an additional benefit of films composed of biodegradable materials, such as telopeptide-free reconstituted collagen. Without compromising their mechanical strength, the loaded films are easily sterilized and become malleable after being hydrated. Film dressings are designed for extended use, typically seven days, but the duration depends on several wound characteristics. The wound’s size influences the frequency of dressing changes; more extensive wounds may require more frequent changes due to increased exudate production. The type of wound also plays a significant role, with heavily exuding wounds needing more regular dressing replacement than dry or minimally exuding wounds. Additionally, the location of the wound affects how long a film dressing can remain effective. Wounds in areas subject to movement or friction may necessitate more frequent changes to maintain proper adhesion and prevent dressing failure [89,95,96,97].

Rathod et al. [98] developed a Col film loaded with *Calendula* flower extract (CFE) as a safe and efficient wound dressing. Physicochemical characteristics such as water absorption capacity, total phenolic and total flavonoid compounds, antioxidant activity, infrared spectra, biological degradation, moisture level, differential scanning calorimetry, and sterility were assessed for the Col film produced by the solvent casting method. The formulation’s collagen’s triple-helical shape was verified using atomic force microscopy and gel electrophoresis. The safety and effectiveness of the created formulation were studied using in vitro cell culture tests such as the MTT assay, scratch assay on the 3T3 Swiss albino cell line, in vitro hemolysis research, and in vivo wound healing animal investigation on Wistar rats. The film produced was found to be noncytotoxic by an in vitro cell cytotoxicity assay. Wistar rats used in an in vivo investigation demonstrated quicker wound healing than the placebo and non-treated groups. On day 21, the CFE-loaded Col film-treated wounds had a considerably higher rate of wound contraction (92.18 ± 1.2%) than the placebo-treated group (62.5 ± 5.1%), control group (56.25 ± 4.1%), and marketed product-treated group (68.75 ± 6.1%). *Calendula* flower extract was added to the manufactured biodegradable collagen film, which proved stable, safe, and efficient as a dressing for wound healing.

**Figure 3:**
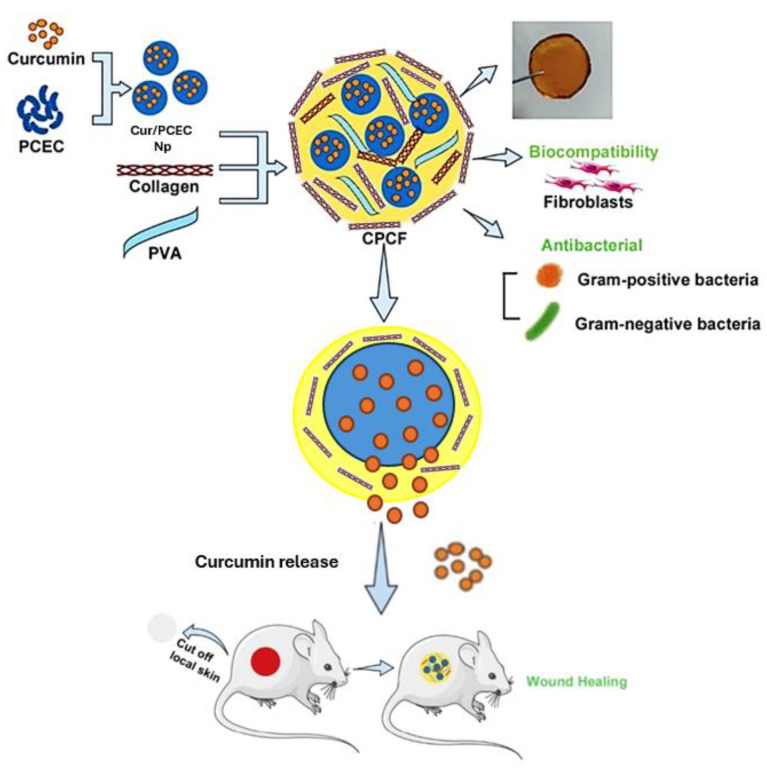
Collagen–PVA composite film loaded with curcumin nanoparticles for sustained drug release and wound healing. Adapted under an open-access license [99].

Leng et al. [99] produced Col–PVA composite films (CPFC), which included curcumin/poly(ε-caprolactone)-poly(ethylene glycol)-poly(ε-caprolactone) (Cur/PCEC) nanoparticles as a wound dressing, allowing them to air dry (Figure 3). Being an essential part of the skin, collagen has low antigenicity and is more biocompatible than other natural polymers. First, the PCEC polymer was used as a carrier to deliver curcumin by forming curcumin nanoparticles. Then, the Cur/PCEC nanoparticles were incorporated with collagen and PVA to build a composite film used as a wound dressing for skin healing. Collagen’s ability to form films was enhanced by the addition of PVA, which made it possible for the film to stick to the wound surface more effectively. Cur/PCEC nanoparticle-loaded Col–PVA composite films exhibited potent antibacterial activity and prolonged drug release behavior. The wound treated with Col–PVA composite films had no secretion, redness, edema, or suppuration. On day 6, the collagen-based films group had the fewest inflammatory cells. At the same time, collagen fibers and new blood vessels developed, showing that they were biocompatible and had an anti-inflammatory impact. Because collagen and platelets interact, transforming growth factor-β1 (TGF-β1) was substantially expressed in the collagen-based films group, significantly enhancing keratinocyte proliferation and epidermal remodeling.

### 5.2. Sponges

Collagen sponges (CSps) serve as effective wound dressings primarily due to their highly interconnected and porous structure, which allows them to absorb blood and wound exudate. This absorption capacity reduces the risk of skin maceration, facilitating hemostasis and promoting effective wound healing. The CSp’s unique architecture provides air and water vapor permeability, which helps maintain a moist wound environment while providing thermal insulation. These characteristics make CSp suitable for various wound types, including partial or full-thickness wounds, heavily exudating wounds, and transplant donor sites. The stability and mechanics of the CSp shape require improvement. The frequency with which CSp dressings must be changed depends mainly on their saturation level. Once the sponge is saturated with absorbed exudate, replacement is required to maintain its efficacy. The replacement schedule can vary significantly, ranging from once or twice per week to as often as daily, depending on the volume of exudate and the specific wound characteristics. Regular assessment of the wound and the sponge’s saturation is essential to determine the optimal changing frequency, ensuring a conducive environment for healing and preventing complications [92,100,101,102,103].

**Figure 4:**
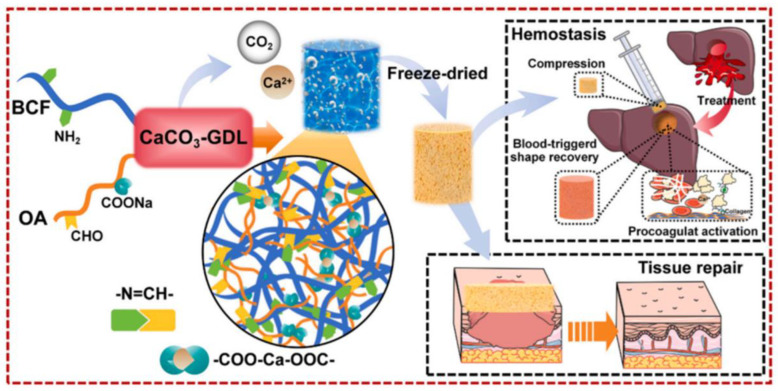
The BCF/OA shape memory sponges’ design explanation. Reprinted with permission from [104]. Copyright © 2024, Elsevier.

Zhang et al. created a bioactive shape memory sponge to meet the urgent need for short-term fast hemostasis and long-term wound healing in noncompressible hemorrhage patients (Figure 4). Using oxidized alginate (OA) and biomimetic collagen fibril (BCF) as a natural backbone, together with an inert calcium supply (Ca) from the CaCO_3_-GDL delayed gelation process, a composite sponge was produced by spontaneously producing pores and double crosslinking under moderate circumstances. The optimized BCF/OACa sponge achieved a physical hemostatic mechanism by efficiently absorbing blood following compression and returning to its initial condition in 11.2 ± 1.3 s. The composite sponge enhanced physiological coagulation by encouraging the adhesion and activation of platelets by BCF and strengthening endogenous and external hemostatic pathways by Ca^2+^. In rat liver damage pick and perforation wound models, the composite sponge decreased bleeding volume and shortened hemostasis time compared to the conventional PVA expanding hemostatic sponge. Furthermore, it promoted wound healing by inducing fibroblast migration and differentiation. It encourages the regeneration of granulation tissue and is biodegradable with a minimal inflammatory reaction [104].

**Figure 5:**
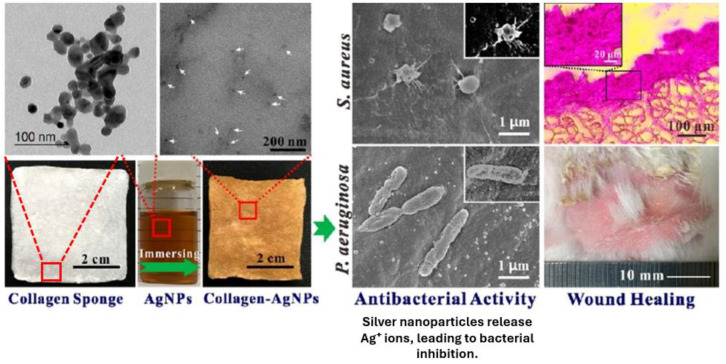
Composite sponge dressings made of Col-AgNPs employed to promote wound healing. Adapted with permission from [105]. Copyright © 2018, American Chemical Society.

Ge et al. [105] prepared a Col-AgNP composite sponge for wound treatment by immersing the collagen sponge in an aqueous solution of silver nanoparticles (AgNPs) (Figure 5). Dialdehyde xanthan gum (DXG) was utilized as a stabilizing and reducing agent to create AgNPs, and it also served as a crosslinking agent to increase the collagen sponge’s stability. Collagen’s inclusion in the Col-AgNP composite sponge decreases the damaging effects of AgNPs on cells. Col-AgNP composite sponges possess a dense porous structure, reduced water vapor permeability, a slower rate of swelling, and superior moisture retention properties compared to collagen sponges. Col-AgNP composite sponges’ tensile strength and elongation at break improved dramatically under dry and swelling conditions. The Col-AgNP composite sponges were gel-like and retained good toughness upon complete swelling. Physical extrusion can simply extract the absorbed free water, which can then remain intact. It possesses a form memory function and may swell repeatedly. CSp with AgNPs had superior antibacterial permeability and antibacterial activity. Furthermore, Col-AgNP composite sponges exhibit strong hemocompatibility and biocompatibility, accelerating collagen deposition and healing full-layer burn wounds, leading to smooth, regenerated skin free of scarring.

Increasing evidence that local nitric oxide (NO) administration might significantly enhance the healing process of wounds has prompted the manufacturing of wound dressings that can topically release NO. Póvoa et al. explored the production of a self-expandable hydrolyzed collagen sponge that releases NO and is charged with S-nitroso glutathione (GSNO), an endogenous NO donor. It was demonstrated that when water is absorbed to a swelling degree of 2300 weight percent, cold-pressed and GSNO-charged CSp (CSp/GSNO) self-expand to its original 3D shape, causing the release of free NO. According to an animal model, exudate uptake by CSp/GSNO releases greater NO concentrations within the inflammatory phase. It gradually reduces NO doses at later stages of the healing process, where wounds are treated topically with compressed CSp/GSNO. In addition, the mRNA expression levels of transforming growth factor beta (TGF-β), stromal cell-derived factor 1 (SDF-1), insulin-like growth factor-1 (IGF-1), monocyte chemoattractant protein-1 (MCP-1), murine macrophage marker (F4/80), nitric oxide synthase (iNOS), and MMP-9 significantly increased in treated animals. On days 7 and 12, immunohistochemistry was used to evaluate cluster differentiation 31 (CD31), vascular endothelial growth factor (VEGF), and F4/80 levels in the cicatricial tissue. These findings suggest that a topical application of NO promotes leucocyte, macrophage, and keratinocyte migration and infiltration into the injured tissue, along with neovascularization and collagen deposition, all of which are linked to a faster rate of wound healing. Therefore, self-expandable CSp/GSNO could be a new biocompatible and effective dressing for applying NO topically to wounds [106].

### 5.3. Hydrogels

Collagen (Col) hydrogels are crosslinked scaffold materials with high water content, typically between 70% and 90%, making them exceptionally well suited for managing dry wounds. Their semi-permeable nature allows for the regulated exchange of both liquids and gases, which facilitates the process of necrotic tissue autolysis and promotes adequate wound debridement by maintaining a consistently moist environment. One of the key advantages of Col hydrogels is their soft and adaptable texture, which enables easy trimming for a precise fit to the wound site. Their inherent transparency further enhances their utility by allowing healthcare providers to monitor wound healing progress without requiring frequent dressing removal. These hydrogels exhibit non-adherent properties, ensuring they do not leave residue behind or cause secondary tissue damage upon removal. This feature, combined with its gentle cooling effect, can help significantly reduce post-surgical inflammation and pain, providing a soothing experience for the patient. Because of their low immunogenicity and unique benefits in accelerating cell proliferation and vascularization, granulation tissue development, wound contraction, and preventing scarring, collagen-based hydrogels have emerged as attractive wound dressings. Through chemical and physical crosslinking methods, collagen hydrogels can be engineered to exhibit a range of mechanical strengths, allowing them to be tailored to meet the specific needs of different skin injuries. To ensure optimal wound hydration and healing conditions, hydrogel dressings should typically be replaced every 1 to 3 days, depending on the moisture level of the wound [107,108,109,110].

Lei et al. [111] generated burn wound dressings by combining human-like collagen (HLC), hyaluronic acid (HA), and carboxylated chitosan (CCS), followed by utilizing glutamine aminotransferase (TG) to act as a crosslinking agent (Figure 5). The concentration of human-like collagen strongly impacts the hydrogel’s tensile strain, pore size, and tensile elastic modulus. Col hydrogels were more hydrophilic when HA and CCS were added, producing HLC-HA-CCS hydrogel with superior water retention capabilities and a suitable moisture vapor transfer rate. The HLC-HA-CCS hydrogel’s interconnected porosity network demonstrated an exceptional bacterial barrier function by preventing bacteria from contacting the wounds through the dressing. Also, it can stimulate migration, adhesion, and cell proliferation, considerably accelerate the recovery of deep second-degree burn injuries, and decrease the production of scars in a rabbit burn model. As a result, the HLCHA-CCS hydrogel dressing may aid in healing burn wounds.

To overcome the absence of antibacterial characteristics and bioactivity of PVA hydrogel, Zhang et al. [112] created a new ternary hydrogel system made of PVA, CS, and Col using a simple physical crosslinking technique. The use of PVA hydrogel as a wound dressing is restricted due to delayed wound healing brought on by its shortage of antibacterial properties and bioactivity. To overcome these obstacles, we used a simple physical crosslinking technique to create a new ternary hydrogel system made of PVA, CS, and Col. The synthesized PVA/CS/Col hydrogel demonstrated exceptional biocompatibility, high mechanical strength, and outstanding antibacterial activity based on a reasonable design and laboratory confirmation. Moreover, it demonstrated an ability to effectively heal tissue defects in a rat model 14 days post-surgery, achieving a wound healing rate of 98.8%, significantly exceeding that of other groups. Histological staining indicated that the healing wound tissue had a thicker epidermis and increased hair follicle structures, effectively repairing wound defects. As a result, the hydrogel’s ease of synthesis on a broad scale, accessible cost, and great repairability suggest it has the potential for wound repair.

**Figure 6:**
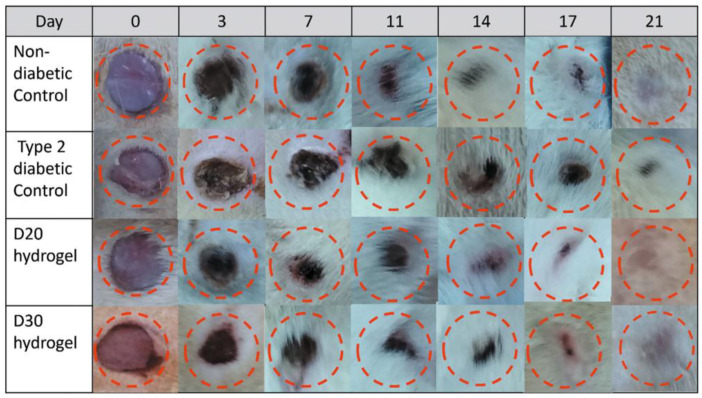
Photographs show how collagen-PU-dextran semi-IPN hydrogels (D20 and D30) affected wound closure in type 2 diabetic rats over 21 days. The type 2 diabetes control group and the non-diabetic control group (*n* = 6) received physiological saline treatment (0.9 wt.%/v.%). The diameter of the orange dotted circle is 12 mm. Reprinted with permission from [113]. Copyright © 2024, John Wiley and Sons.

Aguayo-Morales et al. [109] created biomatrices made of semi-interpenetrated collagen, polyurethane (PU), and dextran polymer networks to improve the wound-healing process. With an impressive crosslinking index of up to 94% facilitated by hydrogen bonding, semi-interpenetrated hydrogels incorporating dextran at levels of 10%, 20%, and 30% demonstrated porous interconnections between collagen fibers and entrapped dextran granules (Figure 6). These hydrogels exhibited notable enhancements in swelling, mechanical characteristics, and hydrolytic and proteolytic breakdown resistance. Several cell types’ viability increased significantly after 24 h, including RAW 264.7 cells, human peripheral blood mononuclear cells, and dermal fibroblasts. After 72 h, these hydrogels also showed a higher release of interleukin-10 and converting growth factor-beta1 while suppressing the release of tumor necrosis factor-alpha and monocyte chemotactic protein-1. Additionally, upon topical administration, these hydrogels accelerated the diabetic rats’ wound-healing process. Notably, wound closure was enhanced in just 21 days by the biomaterial containing 20% dextran (D20). These results demonstrate the potential of the D20 hydrogel, which has biological and physicochemical characteristics that promote wound healing by preventing inflammation and fibrillogenesis while being safe for skin application.

**Figure 7:**
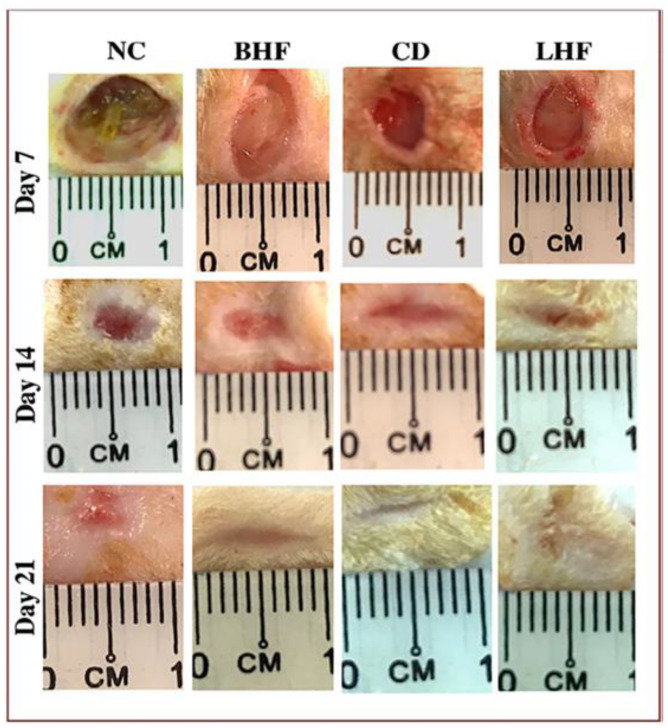
Illustrations of wounds subjected to NC (negative control), CD (commercial dressing), BHF (blank hydrogel film), and LHF (loaded hydrogel film) after 0, 7, 14, and 21 days after wounding. Reprinted with permission from [114]. Copyright © 2021, Elsevier.

Rezvanian et al. [114] created a new hydrogel film wound dressing incorporating sodium alginate and pectin loaded with Simvastatin, which has multifunctional capabilities. This study looked at the in vivo effectiveness of the created wound dressing in a type I diabetic wound model. Experiments were conducted on male Wistar rats for 21 days. Following intraperitoneal injection of streptozotocin (50 mg/kg), the animals developed diabetes. They were subsequently randomly assigned to various groups treated with negative control (NC), commercial dressing (CD), blank hydrogel film (BHF), and loaded hydrogel film (LHF). Animals were euthanized on days 7, 14, and 21 after wounding, and the tissue from the wounds was taken for study. Measurements of vascular endothelial growth factor A, hydroxyproline test, hematological and histological examination, and wound healing rate were recorded. Following 21 days of therapy, the wound dressing considerably (*p* < 0.05) healed the injured region, and the wound closed about 99% of the time without causing any adverse systemic effects (Figure 7). A qualitative histological study showed increased collagen deposition and re-epithelialization. Additionally, the group exposed to the hydrogel film coated with simvastatin exhibited an enhanced rate of angiogenesis and collagen production, according to the data. Because of its pro-angiogenic action, faster re-epithelialization, and higher collagen deposition, the produced film has the potential to accelerate the healing of diabetic wounds significantly.

### 5.4. Nanofibers

Collagen (Col) nanofibers are electrospun wound dressings designed with medium water vapor permeability, effectively regulating moisture levels by preventing excessive exudate accumulation and reducing the risk of wound dehydration. These nanofibers are an advanced wound care material that balances fluid retention and controlled evaporation, which is crucial for maintaining an optimal healing environment. Collagen-based nanomaterials are three-dimensional biomaterials with nanoscale dimensions ranging from 1 to 100 nm. They can be fabricated using various techniques, including electrospinning, melt-blowing, self-assembly, template synthesis, and phase separation. Among these, electrospinning is the most widely employed method due to its simplicity, cost-effectiveness, and ability to produce uniform nanofibrous structures that closely mimic the extracellular matrix (ECM). Collagen nanofibers possess high porosity and a large specific surface area, significantly contributing to wound healing by enhancing cell adhesion, migration, gas exchange, and proliferation. Their nanostructured architecture provides a supportive scaffold for cellular interactions, promoting tissue regeneration and accelerating the healing process. Additionally, collagen nanofibers’ controlled degradation and biocompatibility further enhance their effectiveness in wound management, making them promising biomaterials for various skin injuries and tissue engineering applications [115,116,117,118,119].

**Figure 8:**
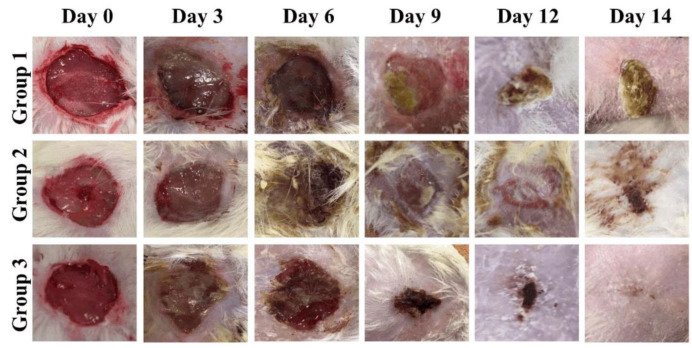
Representative images of skin wounds taken on different days (0–14) show how nanofibers (NFs) affect in vivo wound healing. Group 1 (control), group 2 (PLGA/PMMA/Col/gly (F7)), and group 3 (PLGA/PMMA/Col/gly/SCLE (F11)) are the groups that were analyzed. Reprinted from an open-access source [120].

*Syzygium cumini* leaf extract (SCLE), Col, Gly, poly(lactic-co-glycolic acid) (PLGA), and poly(methyl methacrylate) (PMMA) were used by Abdelazim et al. [120] to create electrospun nanofibers as a scaffold for topical wound treatment with enhanced effectiveness. Conditions for electrospinning were adjusted to produce homogeneous, nanoscale fibers with smooth surfaces. The morphology and swelling behavior of the nanofibers that were produced were examined. Additionally, agar-well diffusion was used to evaluate the nanofibers’ antibacterial efficacy against human and multidrug-resistant microorganisms. The findings showed that, in comparison to the same concentrations of the plain extract, nanofibers containing *Syzygium cumini* extract at concentrations of 0.5 and 1% *w/v* demonstrated stronger antibacterial activity against the tested Gram-positive (i.e., *Staphylococcus aureus*, *Candida albicans*, *Candida glabrata*, and *Bacillus cereus*) and Gram-negative (i.e., *Salmonella paratyphi* and *Escherichia coli*) pathogens. Additionally, throughout 14 days, Wistar rats’ in vivo wound healing was assessed. According to in vivo findings, group 1 was treated with cotton gauze and served as a control; group 2 was treated with PLGA/PMMA/Col/gly (F7); group 3 was treated with PLGA/PMMA/Col/gly/SCLE (F11). As shown in Figure 8, on day 3, the F11-treated group demonstrated 26% wound closure, while the control group treated with cotton gauze displayed only 3.5% wound closure. Compared to the control group, animals treated with F7 and F11 NFs showed remarkable wound healing, with full closure occurring between days 12 and 14. The synergistic impact of SCLE in the F11 scaffold led to faster wound healing, evidenced by the statistically remarkable alteration in wound closure among the control group and F7 and F11 groups. These results demonstrate how artificial nanofibers may treat topical acute wounds and accelerate wound healing.

**Figure 9:**
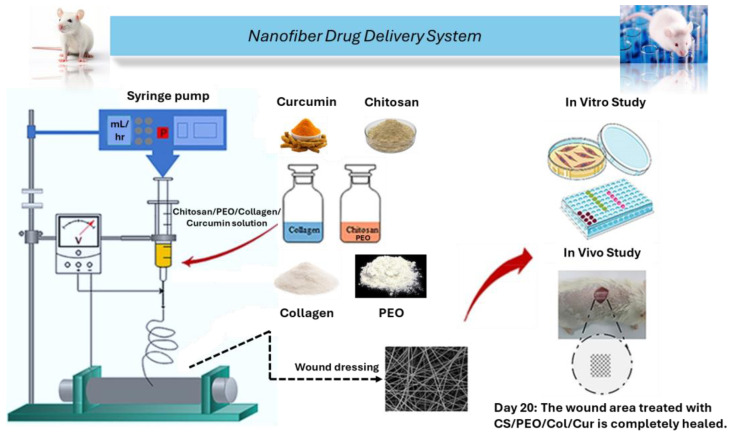
The application of CS-PEO-Col nanofibers to wound healing. Adapted with permission from [121]. Copyright © 2021, American Chemical Society.

Jirofti et al. [117] conducted blend electrospinning to create a chitosan-poly(ethylene oxide)-Col (CS-PEO-Col) nanofiber wound dressing that contained Cur (Figure 9). The Col-prepared nanofiber scaffold has outstanding biocompatibility and offers an area for Cur loading. Col’s weak mechanical qualities can be successfully improved by blending them with CS-PEO. The hydrophilicity of CS-PEO-Col nanofibers loaded with Cur is higher than that of CS-PEO-Col nanofibers alone. As the concentration of Cur increased, there was a significant rise in both hydrophilic characteristics and water absorption capacity. The findings of the cell experiment demonstrated that the CS-PEO-Col nanofibers loaded with Cur were sufficiently biocompatible and did not cause cytotoxicity to human dermal fibroblasts (HDFs). The Alamar blue test assessed the viability of HDF cells grown on CS/PEO/Col nanofibers (with or without) for one to three days. For Col and CS/PEO/Col nanofibers, the cell viability was 107% ± 13% and 111% ± 10%, respectively. When compared to CS/PEO/Col, the untreated control sample, the Cur-loaded CS/PEO/Col nanofibers demonstrated greater cell vitality (127% ± 11% for 5% Cur, 132% ± 6% for 10% Cur, and 115% ± 6% for 15% Cur). The highest cell viability was recorded for CS/PEO/Col/Cur 10% nanofibers. According to the results of the in vivo investigation, CS-PEO-Col nanofibers loaded with Cur may decrease the risk of infection, increase the closure of the full-layer wound in the rat model, and accelerate wound healing. Nanofiber membranes (CS/PEO/Col and Cho/PEO/Col/Cur 15% groups) were applied to rats with wounds made between their shoulders for 1, 5, 10, 15, and 20 days before the nanofibers were taken out. Compared to untreated groups, the Cur-loaded CS/PEO/Col nanofibers (CS/PEO/Col/Cur 15%) significantly accelerated the healing of the wound region.

To enhance their therapeutic effects, Col nanofiber dressings can also be loaded with medications and other bioactive molecules, including metal-based nanoparticles (e.g., Ag, Cu, and Zn nanoparticles), plant extracts (e.g., aloe vera and curcumin), vitamins, growth factors, and antibiotics (e.g., ciprofloxacin, metronidazole, gentamicin, and norfloxacin). These therapeutic compounds can considerably enhance the biological functions of nanofibers [122,123,124,125,126,127]. Ghorbani et al. [128] developed electrospun Col/PCL/zein hybrid nanofibers filled with aloe vera and zinc oxide nanoparticles (ZnO NPs) for wound treatment applications. Increased tensile strength is one aspect of the nanofibers’ mechanical performance. In vitro biodegradation studies showed a weight reduction exceeding 30% for Zein/PCL nanofibers with a 90:10 ratio, 42% for those with an 80:20 ratio, and 54% for the 70:30 ratio, suggesting that a higher proportion of PCL facilitated this process biodegradation. Compared to plain nanofibers, the cytotoxicity analysis of co-loaded nanofibers showed enhanced fibroblast cell adhesion and proliferation, indicating superior biocompatibility and non-toxicity. The plain nanofibers had no inhibition effects against Gram-negative *Escherichia coli* and Gram-positive *Staphylococcus aureus*, according to in vitro antimicrobial studies. In contrast, the drug-co-loaded hybrid nanofibers showed large inhibition zones against both strains of bacteria. These scaffolds are ideal for wound dressings since the bioactive substances load (ZnO NPs and Aloe vera) enhanced biocompatibility and antibacterial properties, indicating a synergistic impact of dual drug-loaded nanofibers.

## 6. Fabrication Techniques for Collagen Dressings

Due to the many application requirements, including wound healing and exudate control, manufacturing dressings for chronic wound applications—such as VLU, PU, DFU, and burns—is challenging. A significant advance has been made in developing wound dressings from simple natural materials to sophisticated designs that prioritize moisture management. Active agents can be delivered using wafer and freeze-dried dressings, which have shown promise in improving wound healing [129].

Freeze-drying is a typical biofabrication technique for biopolymers occurring naturally in vivo. It represents a popular method for treating materials made from natural polymers because it eliminates the solvent without affecting the polymer’s structure. Additionally, it is one of the few methods that can create macropores in natural polymer structures alongside phase separation. Furthermore, it makes it possible to produce three-dimensional sponges with great porosity that can serve as scaffolds for tissue engineering [130,131]. The term “lyophilization”, which means “loves the dry state”, is frequently used to describe freeze-drying. However, it does not apply to the freezing process itself. Although lyophilization and freeze-drying are frequently employed interchangeably, freeze-drying appears to be a more appropriate phrase. When the sample is fully frozen and put under low pressure, the solvent—typically distilled water—sublimates, enabling the ice to go directly from solid to gas without going through the liquid phase. Freeze-drying involves three distinct and interconnected stages: freezing, primary drying (sublimation), and secondary drying (desorption). Each step is built on a separate set of fundamental ideas, which substantially influence the result [132,133]. Col is a popular biomaterial used in medicine to make freeze-dried sponges. To meet the needs of tissue engineering applications such as wound healing, the characteristics of the resultant Col-based sponge may be modified using a variety of additives, which are classified based on their chemical composition and properties. The initial collagen solution may be supplemented with natural polymers (e.g., CS) as well as synthetic polymers (e.g., polycaprolactone (PCL)) to create composite microstructure hydrogel before the freeze-drying procedure. The final scaffold’s characteristics change as additives are added to the initial solution [131,134,135,136].

Because of its simple and low-cost operation, the solvent casting process is a reliable, preferred, and widely utilized casting technology. In the solvent casting method, a polymer and plasticizer are dissolved, the solution is spread out on a substrate, and the solvent is removed. This results in plasticizer molecules’ intercalation and the polymer chains’ molecular orientation, which form a film. This process involves dissolving the polymer or polymers and plasticizer in a volatile solvent, such as water, ethanol, acetone, or a mixture of solvents. If included, a drug can be either dissolved or suspended in the solution, which is then poured into a mold and allowed to dry. [137,138]. Numerous benefits of the solvent casting technique include enhanced physicochemical characteristics, simplicity in use, economical processing, and sufficient thickness consistency. Because the temperatures required to remove the solvent are very low, this method is also used to create films that include heat-sensitive active pharmaceutical ingredients (APIs). Adjusting processing parameters, such as solvent casting duration and temperature, makes it possible to tailor the film’s mechanical and optical characteristics, producing films with excellent optical clarity and porosity [139,140].

The solvent casting process offers several desirable benefits for wound healing, including flexibility, cost-effectiveness, transparency, porosity, and impermeability. Transparency provides a more precise evaluation of the wound, eliminates the necessity for regular wound dressing disposal, and reduces infection rates and trauma while changing the dressing. Oxygen permeability and porosity, known as moisture vapor, prevent anaerobic bacteria from growing and transferring water vapor underneath the dressing to the surrounding environment. Impermeability is a physical barrier against water and germs and prevents bacterial development in response to moisture and microorganisms. Additionally, the dressing may promote autolytic debridement and a moist wound environment [141,142]. Col films made by solving casting methods are widely employed in tissue engineering because of their exceptional film-forming qualities. Nevertheless, several drawbacks to the physicochemical characteristics of Col films restrict their use in wound dressings. Poor handling characteristics of collagen films can be an issue, particularly following contact with exudate. Therefore, to enhance Col’s characteristics, appropriate physical or chemical adjustments are required [143].

Electrospinning utilizes electrostatic forces to produce polymer fibrillar structures, with fiber sizes varying from nano to micrometers. The four main parts of the electrospinning apparatus are a metallic collector, a digital syringe pump, a spinneret (syringe), and a high-voltage power supply [144]. Polymer fibers form when high voltages are applied between the syringe needle and the collector, overcoming the surface tension of the polymer solution. A polymer jet may occur because the drop at the needle tip becomes statically charged. As the voltage rises, the formation of charge on the drop surface changes the drop’s structure in a conic form. A continuous mat of dry polymer fibers is then produced when the solvent evaporates, and the polymer jet is extended by electrostatic repulsion and eliminated towards the collector. Electrospinning parameters—including applied voltage, flow rate, tip-to-target distance, collector shape, needle gauge, and the characteristics of the precursor solution (such as polymer concentration, solvent, surface tension, conductivity, and viscosity)—regulate the morphology, diameter, and orientation of individual fibers. Additionally, environmental factors like temperature and humidity affect fiber properties [145,146].

Electrospinning nanofiber membranes’ porous shape can replicate the ECM of real tissue, promoting the adhesion, proliferation, migration, and differentiation of epithelial cells. The increased specific surface area allows for the potential multi-functionalization of nanofibers, facilitates drug loading and release, and efficiently absorbs exudate from injured sites to prevent bleeding. Furthermore, the electrospinning nanofiber films’ random mesh arrangement and high porosity of 60% to 90% promote cellular respiration, stop excessive water loss, and keep the trauma surface moist. They also prevent external microbiological invasion and the growth of granulation tissue from the trauma surface into the dressing. Because of these benefits, electrospinning nanofibers might be the most preferred method of wound care [147,148]. Recent studies have shown that there are three types of electrospun mats for wound care: (a) pure Col, (b) Col mixed with synthetic and/or natural polymers, and (c) Col-coated fibers that initially were produced with another polymer. Pristine Col fibers have some drawbacks regarding physicochemical characteristics, such as limited temperature stability, poor solvent stability, and low mechanical strength, which might restrict their uses. Therefore, for the possible medicinal use of pure Col nanofibers, efforts have been focused on improving such properties and finding novel Col sources [149]. For use in skin tissue engineering, Col blends containing natural and synthetic polymers like HA, elastin, silk fibroin (SF), polyethylene oxide (PEO), PCL, poly (L-lactic acid) (PLLA), and PVA have been developed. The diameter, porosity, hydrophilicity, and competition of the materials may all impact the growth of cells on the nanofibrous membranes [26,150]. Functional groups greatly influence polymer surface modification. Collagen may be grafted onto scaffold fibers to improve hydrophilicity and ECM replication by forming chemical bonds with functional groups that are activated carboxylic. Carboxyl groups may be activated using 1-ethyl-3-(3-dimethyl aminopropyl) carbodiimide (EDC) to produce highly reactive O-acylisocarbamide compounds. N-hydroxysuccinimide (NHS) is utilized in conjunction with EDC to increase grafting efficiency. It increases the reaction rate while preventing the development of side products. Col-grafting using EDC/NHS chemistry is another method for producing electrospun materials with the added benefits of polymer mixes [151].

Bioprinting is a contemporary technique that may be used to manufacture tissue or organ carriers and cell scaffolds using polymers that imitate tissue characteristics and provide a natural environment for cell growth. Besides being biocompatible, the bioink must promote cellular differentiation, proliferation, and adhesion while avoiding cytotoxicity. To promote tissue regeneration and gradually transfer burden to newly formed tissue, the scaffold should degrade at the same pace that new tissue is created. A 3D-bioprinted scaffold that mimics the native tissue ECM requires a bioink with balanced rheological, biological, chemical, and structural properties. For the scaffold to promote tissue regeneration, maintain the integrity of its structure, and blend in with the host tissue, these parameters must be precisely modified to meet the requirements of the target tissue. Topologies and bioinks must be selected to functionally customize 3D bioprinting scaffolds for human tissues [152,153].

ECM and Col-based polymers are among the many different types of polymers that are utilized, both natural and manufactured. The best instruments for producing accurate structures from the combination of Col hydrogel and cells are bioprinting technologies based on syringe deposition or laser technologies [152]. Col’s unique features of biocompatibility and low immunogenicity make this achievable. The dynamics of this procedure determine how printable Col bioink is; the faster the process, the more accurate the print. Most current research on Col bioprinting and 3D printing identifies the main drawback of Col bioink: its poor mechanical qualities. Utilization of supporting hydrogels is one potential strategy to get over this restriction. The process occurs inside the secondary hydrogel (such as gelatin slurry) when utilizing a supporting hydrogel for 3D bioprinting with Col bioink. This hydrogel serves as a short-term thermo-reversible support (FRESH method—freeform reversible embedding of suspended hydrogels) [154,155]. When Col and alginate are combined to produce a bioink, they encourage the growth of fibroblasts and keratinocytes at the liquid–liquid interface when exposed to air. The cells may proliferate and spread, creating a structure that resembles human skin [156]. A multilayered skin structure was developed by layering keratinocytes using inkjet printing and extruding Col and PCL mesh to generate skin layers on a PDMS microfluidic device. The results show that the bioprinted structure prevents contraction during tissue formation by encouraging cell division and protein release. Skin biomimetic porous scaffolds have been created using a two-step drop-on-demand bioprinting process. Due to its numerous layers, unique coloring, and constant cell distribution, this model largely resembled human morphology [157].

Crosslinking has been utilized to improve mechanical stability, reduce deterioration, and alter the molecular structure of Col [158]. Researchers looked at various methods, such as adding different additives to a Col-based wound dressing, to determine how the components of these additives affected morphology, physicochemical properties, compressive mechanical properties, and cytocompatibility. Crosslinking technique, exposure duration, and crosslinker concentration have also significantly impacted the final sponge characteristics. Numerous crosslinking methods have been created and can be categorized as enzymatic, chemical, or physical methods [131,159]. Physical crosslinking, for example, involves UV radiation and DHT [160]. Chemical crosslinkers include glutaraldehyde (GA), 1,4-butanediol diglycidyl ether (BDDGE), and EDC coupled with NHS and genipin (GNP). In contrast, transglutaminases carry out enzymatic crosslinking throughout the body, creating bonds between collagen monomers, leading to even higher molecular weight molecules [161]. Investigations have also been conducted into possible combinations with other support materials. For instance, Wang et al. [162] demonstrated that the inclusion of HAp significantly increased the composite Col films’ tensile strength. In a different study, Teronová et al. [143] introduced a novel approach to reinforcing Col films by combining Col with partly modified microfibrillar carboxymethylcellulose (CMC), which had never been reported before. Furthermore, while Col-based wound dressings naturally encourage cell attachment and proliferation, including functional molecules such as growth factors in the sponge is beneficial in increasing the potential for regeneration in some situations. The type of biomolecule, its concentration based on the target tissue, and its release rate are significant challenges for scientists studying these functional molecules. Col shows limited responsiveness to certain growth factors, potentially leading to inadequate sustained and delayed release for effective tissue repair applications. Furthermore, using growth factors raises hospital and clinical expenses [163].

## 7. Clinical Applications and Use Cases

Innovative ways are becoming more popular in wound care because of the growing need for customized medication delivery plans to lower dosage frequency. Traditional topical formulations, such as creams, gels, and lotions, are helpful for localized drug administration. Still, they frequently lack controlled release properties, making developing new dosage forms necessary. Traditional topical therapies provide localized effects without controlled release advantages since they mainly rely on passive diffusion into the skin. As a result, there is an increasing demand for innovative topical dosage forms that can deliver medications to wound sites gradually and precisely [164]. Col dressings are commonly used in clinical settings to support wound healing by reducing patient discomfort, preventing infections, and significantly expediting recovery. These dressings play a crucial role in managing chronic wounds such as burns, DFUs, VLUs, and PUs [92,165].

### 7.1. Clinical Application of Collagen Wound Dressings in Diabetic Foot Ulcers

DFUs are a common and dangerous result of diabetes mellitus due to decreased wound healing. The most common feature of DFUs is epidermal ulceration, resulting in tissue integrity loss and ECM degradation. DFUs are primarily caused by neurological and sensory deficiencies, peripheral neuropathy, and vasculopathy, which limit blood circulation. Due to several factors, including poor general health, more prevalent underlying abnormal platelet activity, and growth factor deficiency, which reduces the ability to repair traumatic tissues, patients with DFUs have a high rate of amputation and death [166].

Munish et al. [167] treated patients with DFUs using collagen-based wound dressings. Two groups of fifty patients were formed. Twenty-five patients in the control group received standard saline treatment, whereas twenty-five patients in the experimental group received Col dressings. The bandage was changed once or twice daily for 12 weeks or until the ulcer healed. Two patients in the experimental group cured their ulcers completely after a week of therapy, while twelve patients had much-diminished ulcer areas. The control group showed no ulcer healing; only six patients had a considerably decreased ulcer area. Twenty-one patients in the experimental group had their ulcers fully cured, and their ulcer areas shrank after 12 weeks of treatment. In contrast, only seven patients in the control group fully healed their ulcers, and their ulcer areas shrank. According to this study, Col wound dressings could accelerate the healing process for DFUs.

CS/Col composite hydrogel can stimulate the healing of DFUs. Djavid et al. [168] conducted a study to assess the effectiveness of a novel Col matrix dressing that contains CS and Col hydrogel with a conventional dressing of saline-moistened gauze for wound recovery in patients with neuropathic DFUs that is difficult to heal. Following standard wound care procedures that included debridement, infection management, and offloading, patients were randomized into receiving either a collagen matrix bandage (the study group, receiving the Tebaderm producer) or a saline-moistened gauze treatment (the control group). Measurements were made throughout therapy and at follow-up to determine the number of patients who experienced full healing and decreased DFU size. Sixty-one patients with neuropathic DFUs in all were enlisted. At four weeks, the study group’s average percentage decrease in DFU size was higher than that of the control group (54.5% versus 38.8%, respectively). During the 20-week follow-up, the study group’s full healing rate was substantially more significant than the control group’s (60% versus 35.5%, respectively). In this study, patients with difficult-to-heal DFUs experienced faster healing thanks to the Col matrix dressing. According to further research, it may be possible to reduce the time needed for full healing by using this dressing.

Fleischli et al. [169] employed horse pericardium Col dressings to treat 22 individuals with neurogenic DFUs. Patients had dressing changes every three to four days for twelve weeks or until they healed. According to the findings, the wound area significantly improved in 94% of the patients at an average of 2.9 weeks; the wound size shrank by 52.3% in the fourth week, and 47% of the patients recovered by the 12th week. Col dressings for the pericardium of equine may be a safe and effective therapy for neurogenic DFUs, according to this study.

### 7.2. Clinical Application of Collagen Wound Dressings in Venous Leg Ulcers

The VLU is the most prevalent kind of lower extremity ulcer. The VLU is characterized as a full-thickness skin defect commonly observed in the ankle area, is caused by chronic venous disease (CVD, the range of venous illnesses affecting the lower extremities), and does not heal independently. According to current standards, a VLU is characterized by best practices and the conventional definition of an exposed leg or foot skin lesion that develops in a region affected by venous hypertension, primarily classified into venous blockage and venous valve regurgitation [170].

Romanelli et al. [171] treated patients with VLUs using Col and alginate bandages. Block randomization was used to split 40 patients into two groups. The participants in the control group were given only an alginate dressing, while both treatments were provided to the experimental group. The dressings were replaced two times a week for twelve weeks or until the ulcer healed. After that, the improvement of the granulation tissue, the wound’s size, the dressing’s overall effectiveness, and the dressing’s comfort were assessed and documented. According to the findings, the experimental group’s granulation tissue grew by 65%, but the control group’s only increased by 38%. Patients in the experimental group saw an average ulcer area reduction of 45% after 12 weeks, whereas patients in the control group experienced a reduction of 20%. These findings suggested that Col dressings could effectively encourage the regeneration of granulation tissue and accelerate the healing of VLUs.

Morimoto et al. [172] have created a new Col/gelatin sponge (CGS) artificial dermis that can continuously secrete basic fibroblast growth factor (bFGF) for over ten days. This investigation aimed to assess the safety and effectiveness of CGS impregnated with bFGF in treating VLUs. After debridement, CGS impregnated with bFGF at 7 or 14 mg/cm^2^ was administered to patients with VLUs that had not recovered in at least 4 weeks. The improvement of the wound bed was evaluated 14 days later. A granulated and epithelialized region on day 14 that had a percentage of 50% or more to the initial wound area following debridement was considered to indicate an improvement in the wound bed. To monitor any negative responses associated with the use of CGS, patients were monitored for a total of 28 days following application. Patients recovered quickly from moderate adverse reactions that had a direct causal link to the study therapy. The safety and effectiveness of CGS impregnated with bFGF in treating chronic VLUs were demonstrated, and this is the first-in-man clinical trial of CGS. For persistent skin ulcers, this combined therapy may be a successful treatment.

Mościcka et al. [173] treated patients with venous leg ulcers using a Col gel bandage made from fish skin. Forty-five patients in the control group received just a placebo, whereas 47 patients included in the experimental group received fish skin Col gel dressing treatment. The CIVIQ and Skin-dex-29 measures were used to measure quality of life (QoL) and healing rate (cm^2^/week). The findings demonstrated that the experimental group had more ulcer-healing patients, a faster ulcer-healing rate, and significant improvements in quality of life following therapy. This study has shown that treating VLU patients with fish Col gel dressing enhanced their quality of life and healing process while reducing the intensity of their physical complaints, discomfort, and local skin symptoms.

### 7.3. Clinical Application of Collagen Wound Dressings in Pressure Ulcers

A combination of physiological processes and environmental factors can lead to the development of PUs. More thorough research has been carried out on the conventional hypothesis that the only cause of PU development is tissue ischemia brought on by extended external pressure and shear and/or frictional forces impacting the tissue. PUs can spread further into the periosteum and bone, resulting in localized osteochondritis or osteomyelitis. Secondary infections that can lead to sepsis, skin cancer, or even death can occur in patients with severe PUs [174].

The wound microenvironment is improved by oxidized regenerated cellulose/Col matrix (ORC/Col matrix), which binds and deactivates excess proteases in wound exudates. To evaluate the positive impact of ORC/Col matrix treatment compared to control treatment with a foam dressing, Kloeters et al. [175] assessed the activities and level of plasmin and elastase in wound exudates from pressure sore ulcers. The research included 33 pressure sore patients monitored for 12 weeks following therapy. A foam hydropolymer dressing (TIELLE^®^, Systagenix, Crawley, United Kingdom) was applied to 10 control patients. In contrast, ORC/Col matrix and the foam dressing (TIELLE^®^) were applied to the remaining 23 patients. Wound evaluations were conducted over 12 weeks, with dressing changes occurring twice a week. Photographs of ulcers were taken, and wound exudates were gathered admission at 5, 14, and every 14 days to track changes in appearance and healing rate and conduct biochemical analysis. Elastase and plasmin activity and levels were assessed in wound exudates. The healing rate of pressure sore wounds treated with ORC/Col matrix was significantly faster than that of controls, and this was positively connected with lower plasmin and elastase activity in wound exudates. There were no indications of infection or sensitivity to the ORC/Col matrix.

Piatkowsk et al. [176] mixed Col dressing with foam dressing to treat patients with PUs. Ten patients were split equally and randomly into two groups; the control group only utilized the same foam dressing, while the experimental group used a Col dressing coated in foam. The wound was assessed on days 0, 3, 7, 14, and 21 of therapy. Compared to the control group, the amount of MMP-2 in the wound exudate of the experimental group reduced considerably on the third day. Still, the concentration of MMP-9 declined rapidly and to a greater degree throughout the trial. There was a considerable rise in TIMP-1 and TIMP-2 concentrations on the first and fourteenth days. In the experimental group, the PU healed faster. By the fourteenth day, 40% of the patients’ PUs had healed, compared to 0% in the control group. Only 80% of the patients in the control group witnessed healing on the twenty-first day, compared to all the patients in the experimental group. The trial outcomes demonstrated that using Col dressings in conjunction with foam dressings might significantly reduce the time it takes for PUs to heal.

### 7.4. Clinical Application of Collagen Wound Dressings in Burn Injuries

Burn injuries are categorized based on various factors, including depth, origin, and body injury surface area percentage. The combination of the classifications mentioned above determines the degree of burn damage. There are two types of burns: “partial-thickness” and “full-thickness”. Recovery will be quick (10–14 days), and there is little chance of scarring if the injury is restricted to the epidermis and the outer layer of the dermis (a superficial partial-thickness burn), leaving most of the appendage structures unharmed. Conversely, suppose the burn penetrates more profound levels of the dermis and does more damage to the appendages. In that case, the epithelium will require more time to repair (3–6 weeks), and the possibility of hypertrophic scarring is higher. All skin layers are destroyed with full-thickness burns, and surgery is typically necessary to promote appropriate wound healing [177,178].

Mehta et al. [179] have employed silver-sulfadiazine (SSD)-impregnated CSp (Col-SSD) dressings for managing second-degree patient burns. In a random and equal distribution, 50 patients were split into two groups. The experimental group received treatment with Col-SDD dressings, while the control group received gauze-assisted SSD. According to the findings, by day 7, 22 patients in the experimental group had fully recovered. However, just 14 patients in the control group experienced full recovery, and the experimental group’s mean time to full recovery was considerably shorter than that of the control group. When pain scores on the Visual Analog Scale (VAS) were compared between the two groups on days 2 and 7, patients in the experimental group experienced significantly less pain in comparison with those in the control group, whose pain was mainly attributed to frequent dressing changes. Except for two patients who needed skin-grafting, all experimental group patients showed improved wound healing within seven days of treatment. In the control group, 14 patients exhibited better wound healing; the rest required extended care or skin-grafting. As a result, employing a Col-SSD dressing for burns may decrease wound healing time, minimize patient discomfort, lessen the frequency of dressing changes with no significant complications, and stop patients from requiring skin grafting treatment.

Ben et al. [180] created a recombinant human collagen hydrogel (RHCH) patch for treating partial-thickness burn patients. Wounds of twenty-one patients were evenly divided along the axis and treated with either a human-CTLA4-Ig gene-transferred pig skin xenotransplant or RHCH. Days 0, 5, 10, 15, and 20 were used to document the state of the wound surfaces. The data show no significant statistical differences in wound healing times between the two groups. With RHCH dressing, the median healing time was 11.00 ± 1.72 days, while a xenogeneic skin dressing was 11.00 ± 0.56 days. The findings mentioned above demonstrated that the authors’ RHCH dressing has potential for clinical use and that its effectiveness in healing partial-thickness burns is not appreciably distinct from that of a transgenic skin xenograft.

Although collagen-based therapies have demonstrated promise in tissue regeneration and wound healing, several drawbacks limit them from being widely used in clinical settings. Different animal sources of collagen can impact the final product’s quality and bioactivity, as can variations in the source and extraction techniques. Inconsistencies in collagen characteristics can also lead to differences in patient outcomes. Collagen dressings alone might not be enough to stop infection, especially in wounds that are polluted or exude a lot. One major drawback in treating such wounds is the absence of inherent antibacterial qualities. Collagen dressings are frequently combined with bioactive substances or antibacterial agents to increase their effectiveness, which makes treatment plans more difficult and raises expenses. Collagen-based products may cause allergic or inflammatory responses in some individuals, especially if made from animal tissues. As a result, their use may be limited in some demographics. Age, underlying medical chronic disorders, and general immunological state are some variables that might significantly affect how a patient reacts to collagen therapies. Depending on how they are made and the conditions surrounding the wound, collagen dressings break down at different rates. While slow deterioration may lengthen treatment times and complicate wound care, rapid degradation may result in inadequate mechanical support [181,182].

### 7.5. Commercially Available Dressings for Collagen Skin Repair

The US Food and Drug Administration has authorized several Col wound dressings for treating acute and chronic wounds. Col wound dressings can be purchased commercially as films, microfibers, sponges, powders, and gels (hydrogels). They are able to be used to treat surgical injuries, lacerations, radioactive wounds, and various acute and chronic conditions, including arteries, veins, diabetes, and PUs [92]. The commercially available Col wound dressings are listed in Table 2.

## 8. Challenges and Limitations

There are several obstacles to advancement in wound healing that must be considered. Wound exudates vary, making finding suitable dressing for all wound types challenging. The ideal option is to develop composite dressings, combining many aspects of modern advancements. By using composite dressings, most of the wound healing process will be considered, resulting in quicker healing time and complete treatment [195].

Despite significant advancements in Col wound dressings over recent decades, several inherent drawbacks have hindered their widespread clinical application. These limitations include low mechanical strength, poor thermal stability, susceptibility to enzymatic degradation, and inadequate skin permeability. Such challenges reduce the durability and effectiveness of collagen-based dressings, highlighting the need for further research and innovation to enhance their performance. Previous studies have demonstrated that combining collagen with other materials can improve its healing properties. However, there is still much to explore in optimizing these formulations for better clinical outcomes. One promising strategy involves incorporating pharmaceutical agents and bioactive compounds into collagen dressings, transforming them into multifunctional wound care solutions that accelerate healing, reduce inflammation, and prevent infections. Additionally, integrating cell-penetrating peptides offers a novel approach to enhancing skin permeability, facilitating the deeper delivery of therapeutic molecules into the wound site. By leveraging these advancements, collagen-based dressings have the potential to evolve into highly efficient, next-generation wound care materials tailored to diverse medical needs [92,97].

Moreover, the difficulty of identifying an optimum Col source remains a challenge. The most prevalent biological reactions to introducing foreign objects in biological systems include localized tissue response, hematologic response, immunological response, and acute/chronic systemic toxicity. This response can facilitate the recruitment of immune cells (such as macrophages and neutrophils) to the wound site, promoting tissue repair and regeneration. However, an excessive inflammatory response may hinder healing and increase the risk of chronic wounds. While collagen and its breakdown products (primarily amino acids) are non-toxic and easily absorbed by organisms, other non-biodegradable components of collagen dressing may have harmful biological effects. Animal-derived Col in medicine presents immunogenic and pathogenic risks despite the infrequency of pathogenic infections following scaffold implantation. Nevertheless, concerns about the possible adverse effects of additional compounds recovered with Col originating from animals and some purity problems persist. In the future, genetically altered cows devoid of prion protein could offer a safe supply of collagen-based products [196,197]. Most collagen-containing products typically comprise collagen I and collagen III. In the future, collagen-II-based materials could be required for specific purposes, such as cartilage engineering. Additionally, there are significant differences of opinion about the potential for collagen-II-containing products to trigger autoimmune responses in humans. Scientists are optimistic that they can make recombinant collagen as pure as our bodies naturally generate in the following years, removing any impurities and the risk of spreading infections or provoking immunological responses. New materials created through biological processes are called recombinant collagens [35,196].

Human Col might be produced industrially using recombinant DNA technology. However, modifications after translation, protein folding, and batch yield issues make this method unlikely to gain interest anytime soon. Additionally, certain recombinant Col polypeptide chains cannot form a stable triple helix structure without sufficient enzyme activity. Consequently, significant efforts are needed to enhance the recombinant system and explore new Col types. There may be different microbial components at the beginning of the extraction process if Escherichia coli and other microbes are fermented to produce the recombinant Col. The following factors should be checked and managed to guarantee that the finished product is a high-purity recombinant Col: its purity, heavy metal and trace element contents, antibiotic residues, host cell protein (HCP), exogenous DNA, endotoxin, and any remaining additives like isopropyl thiogalactoside (IPTG) and imidazole. Immunogenicity concerns should be assessed and confirmed for biological products made using microbial fermentation techniques. The molecular level analysis of recombinant Col’s content and structure is easier than that of animals. Moreover, recombinant Col’s composition is more straightforward than Col obtained from animals, making it easier to purify and eliminate different impurities that frequently cause immunogenicity. Thus, this material has a lesser risk of immunogenicity than similar items. Future research should concentrate on developing Col structurally and physiologically comparable to natural Col. In addition to the source, the extraction conditions may directly influence the production and quality of the finished Col. Depending on its quality, the unrefined Col may need to be purified by downstream processing. Before extraction procedures, pre-treatment measures must also be considered to get high-quality Col [56,57,149]. In more detail, the extraction and purification of collagen are intricate processes; traditional methods involve labor-intensive steps such as tissue cleaning, solubilization, and dialysis, which can extend up to a month, thereby hindering scalability and increasing costs. Moreover, maintaining the structural integrity and bioactivity of collagen during processing (especially during crosslinking and sterilization) is complex, with potential implications for the stability and efficacy of the final product [198,199].

Besides the already mentioned issues, variability in collagen sources, particularly those derived from animal tissues, further complicates standardization, leading to batch-to-batch inconsistencies that affect product reliability. Additionally, regulatory hurdles associated with ensuring the safety and efficacy of collagen-based medical devices add layers of complexity to their development and market approval [16,57]. Financial barriers from the high costs of sourcing quality raw materials and extensive purification and quality control measures contribute to elevated production expenses. These costs are often passed on to consumers, limiting the accessibility of advanced collagen dressings, particularly in low-resource healthcare settings [200]. Addressing these challenges is crucial for the broader adoption and equitable distribution of collagen-based wound care solutions

## 9. Conclusions and Future Directions

Ineffective treatment of acute wounds often results in chronic wounds, which pose a significant dermatological challenge. Developing durable and efficient wound dressings is essential to ensure optimal treatment for individuals suffering from chronic wounds. Various biomaterials have been utilized to create wound-healing dressings, with collagen—a necessary component of the extracellular matrix—standing out due to its biodegradability, biocompatibility, and ability to promote tissue regeneration. Given its wide availability and therapeutic potential, collagen-based wound dressings are promising for future wound management. However, the exclusive use of collagen in wound dressings presents challenges, such as weak mechanical properties, inadequate stability, and a lack of intrinsic antibacterial effects compared to other materials. Researchers have explored various strategies to address these limitations, including crosslinking collagen with additional polymers to enhance mechanical strength and stability and incorporating bioactive or antibacterial agents to improve antimicrobial efficacy.

Recent advancements in collagen-based wound dressings, particularly in hydrogels, foams, and composite materials, have significantly improved their therapeutic potential. These innovations enable better moisture retention, controlled drug delivery, and adaptability to the wound microenvironment, optimizing the conditions for wound healing. Collagen also plays a crucial role in angiogenesis, structural support, and cellular signaling, emphasizing its significance in regenerative medicine. The electrospinning of collagen nanofibers, which mimics the extracellular matrix, has shown promise in enhancing tissue homeostasis, cell adhesion, proliferation, and differentiation. Consequently, three-dimensional (3D) scaffolds composed of collagen nanofibers enriched with bioactive ingredients are anticipated to revolutionize wound healing by providing enhanced regenerative capabilities.

Clinical research has demonstrated the effectiveness of collagen-based wound dressings in managing various chronic wounds, significantly alleviating patient discomfort and accelerating the healing process. Given the variety of collagen dressing formulations available, selecting the most appropriate type is essential based on the specific wound condition. Additionally, continuous updates on commercially available collagen-based wound dressings provide valuable insights into their clinical efficacy and real-world applications.

Integrating innovative technologies into collagen-based dressings will be pivotal in advancing wound care. Developing integrated collagen dressings equipped with remote wound monitoring capabilities and visual indicators for changes in chronic wound conditions will enable real-time assessment and intervention. This approach will facilitate the prolonged and targeted application of affordable dressing solutions, reducing the frequency of dressing changes and minimizing the risk of infection. Moreover, flexible design and manufacturing techniques will allow for late-stage device assembly at the bedside, ensuring adaptability to patient-specific needs.

An emerging perspective in this field involves the incorporation of bioengineered collagen dressings with responsive and stimuli-sensitive properties. Future dressings could release therapeutic agents in response to wound conditions such as infection, pH imbalance, or inflammation. Additionally, 3D bioprinting and tissue engineering advancements may pave the way for personalized collagen dressings tailored to individual wound characteristics, optimizing patient outcomes through precision medicine. Collagen-based devices with easy removability, adaptability to the chronic wound microenvironment, and improved infection control are expected to revolutionize wound care, particularly in out-of-hospital settings. Identifying relevant clinical models for early-stage testing before entering clinical trials will further enhance the efficacy and safety of these next-generation dressings.

Despite the significant progress made in collagen-based wound dressings, there remains substantial potential for further innovation. Future interdisciplinary research should focus on developing high-performance, personalized wound care solutions that combine biomaterials science, nanotechnology, and innovative healthcare systems. By addressing current challenges and leveraging emerging technologies, collagen-based wound dressings have the potential to become a cornerstone of next-generation wound management, improving patient outcomes and quality of life.

## Figures and Tables

**Table 1:** Collagen’s classification based on its molecular structure. Data adapted from [27,28,38,40,41,42].

Type	Molecular Composition	Tissue Distribution	Supramolecular Structure and Organization
I	[α1(I)]_2_α2(I)	bone, dermis, tendons, ligaments, and cornea (represent 90% of the total collagen of the human body)	Fibril-forming collagens
II	[α1(II)]_3_	cartilage, intervertebral disc, notochord, vitreous humor in the eye	
III	[α1(III)]_3_	skin, vessel wall, and reticular fibers of most tissues (lungs, liver, spleen, etc.)	
V	α1(V),α2(V),α3(V)	lung, cornea, bone, and fetal membranes, together with type I collagen	
XI	α1(XI)α2(XI)α3(XI)	cartilage, vitreous body	
IV	[α1(IV)]_2_α2(IV); α1–α6	basement membranes	Basement-membrane collagens
VI	α1(VI),α2(VI),α3(VI)	widespread: dermis, cartilage, placenta, lungs, vessel wall, and intervertebral disc	Microfibrillar collagen
VII	[α1(VII)]_3_	skin, dermal–epidermal junctions, oral mucosa, and cervix	Anchoring fibrils
VIII	[α1(VIII)]_2_α2(VIII)	endothelial cells, Descemet’s membrane	Hexagonal network-forming collagens
X	[α3(X)]_3_	hypertrophic cartilage	
IX	α1(IX)α2(IX)α3(IX)	cartilage, vitreous humor, and cornea	FACIT collagens
XII	[α1(XII)]_3_	perichondrium, ligaments, and tendon	
XIV	[α1(XIV)]_3_	dermis, tendon, vessel wall, placenta, lungs, and liver	
XIX	[α1(XIX)]_3_	human rhabdomyosarcoma	
XX	[α1(XX)]_3_	corneal epithelium, embryonic skin, sternal cartilage, and tendon	
XXI	[α1(XXI)]_3_	blood vessel wall	
XIII	[α1(XIII)]_3_	epidermis, hair follicle, endomysium, intestine, chondrocytes, lungs, and liver	Transmembrane collagens (membrane-associated collagens with interrupted triple helices—MACITs)
XVII	[α1(XVII)]_3_	dermal–epidermal junctions	Multiplexins
XV	[α1(XV)]_3_	fibroblasts, smooth muscle cells, and kidney, pancreas	
XVI	[α1(XVI)]_3_	fibroblasts, amnion, and keratinocytes	
XVIII	[α1(XVIII)]_3_	lungs, liver	

**Table 2:** An overview of commercially available Col-based wound dressings.

Form of Dressing	Composition	Product Name	Advantage	Limitations	Wound Suitable for	Refs.
Gel	Col	CellerateRX	Keep the wound bed wet	Bovine sources and secondary dressings are needed	Partial and full-thickness injuries, including traumatic wounds, surgical wounds, DFUs, and burns	[183]
Gel	Col Polypeptides	Stimulen	Keep the wound bed wet	Bovine sources and secondary dressings are needed	Full- and partial-thickness wounds, including PUs, partial-thickness burns, abrasions	[184]
Gel	Col	Collatek	Keep the wound bed wet	Bovine sources and secondary dressings are needed	Cuts, abrasions, minor wounds, critical sunburns, partial- and full-thickness wounds, venous stasis ulcers, burns of the first or second degree, ulcers that have many etiologies, surgical wounds	[185]
Gel	Type I Col	Collogel	Keep the wound bed wet	Type I bovine collagen, and secondary dressings are needed	Pressure sores, dermal lesions, first-degree burns, donor sites, stretch marks and scar management	[185]
Pad	Col fleece, gentamicin salts	Septocoll E	Activate platelets	Skin response	Full and partial-thickness injuries, including infected wounds and bleeding lesions	[186]
Pad	Col and Ca alginate	FibracolPlus	Keep the wound bed wet	Secondary dressings are needed	Full and partial thickness injuries, such as burns, DFUs, VLUs, or PUs, abrasions, and dehisced surgical incisions	[187]
Pad	Bovine Col, and Manuka Honey	Puracol	No additional debridement is needed	Bovine source and expensive	Full and partial thickness injuries, such as burns, DFUs, VLUs, or PUs, abrasions, and dehisced surgical incisions	[188]
Pad	Type I equine Col	Biopad	Free of collagen degradation products	Equine source, time-consuming, high cost	Full and partial thickness injuries, such as DFUs, VLUs, or PUs, abrasions, and dehisced surgical incisions	[189]
Micro-fibrillar	Col	Avitene	Adequate hemostasis, absorbable, and minimizes adhesions	Bovine sources, high cost	Lacerated wound, surgical wounds	[190]
Sheet	Col	Skintemp^®^ II	Keep the wound bed wet	Bovine sources, limited shelf life, potential for infection	Blisters, second-degree burns, arterial, venous, diabetic neuropathic, pressure ulcers, superficial secondary trauma, dehisced surgical wounds, and abrasions	[191]
Sponge	Type I bovine deep flexor tendonCol	Helistat	Effective hemostasis, absorbable	Bovine sources require a dry field	Surgical wounds	[192]
Powder	Bovine cartilage	Catrix^®^	Biodegradable, decreasebleeding	Bovine sources, secondary dressings are needed	Stasis ulcers, DFUs/PUs, first- and second-degree burns, wounds following surgery, cuts, abrasions, irritations, radiation dermatitis, and partial-thickness wounds	[193]
Cellularmatrix	Col,polycarbonatemembrane	Apligraf	Resorbable	Bovine source, expensive, and not suitable for infected wounds	Full and partial thickness damage, such as VLUs and DFUs	[194]

## Data Availability

Not applicable.
